# Carbon Nanomaterials as Antibacterial Colloids

**DOI:** 10.3390/ma9080617

**Published:** 2016-07-25

**Authors:** Michael Maas

**Affiliations:** Faculty of Production Engineering, Advanced Ceramics, MAPEX—Centre for Materials and Processes, University of Bremen, Bremen 28359, Germany; michael.maas@uni-bremen.de; Tel.: +49-421-218-64939

**Keywords:** carbon, nanotubes, graphene, fullerene, diamond, nanomaterial, environment, bacteria, toxicity, antibacterial

## Abstract

Carbon nanomaterials like graphene, carbon nanotubes, fullerenes and the various forms of diamond have attracted great attention for their vast potential regarding applications in electrical engineering and as biomaterials. The study of the antibacterial properties of carbon nanomaterials provides fundamental information on the possible toxicity and environmental impact of these materials. Furthermore, as a result of the increasing prevalence of resistant bacteria strains, the development of novel antibacterial materials is of great importance. This article reviews current research efforts on characterizing the antibacterial activity of carbon nanomaterials from the perspective of colloid and interface science. Building on these fundamental findings, recent functionalization strategies for enhancing the antibacterial effect of carbon nanomaterials are described. The review concludes with a comprehensive outlook that summarizes the most important discoveries and trends regarding antibacterial carbon nanomaterials.

## 1. Introduction

Carbon is able to form different allotropes based on the formation of either sp^2^ or sp^3^ bonds between the individual carbon atoms. These configurations are especially significant at the nanoscale where each allotrope exhibits unique properties. Applications of carbon nanomaterials include their utilization in composite materials, as biomaterials, as drug carriers or as materials for energy storage. Most notably, carbon nanotubes (CNT) and graphene are studied for their vast potential in electrical engineering. Concurrently, albeit to a lesser extent, investigations on the other allotropes of nanocarbon like fullerenes and nanodiamond are steadily gaining momentum. With the increasing prevalence of carbon nanomaterials (CNMs) in nanotechnology, the interaction with living tissue and their fate in organisms as well as consequences for the environment have come into focus. Most allotropic forms of carbon, graphene oxides, carbon nanotubes and fullerenes were soon identified as moderately toxic to most living cells, both for eukaryotic and especially for prokaryotic cells [1,2]. One reason for their toxicity might be their hydrophobic character, which enables penetration of cell membranes [3]. Several studies reported the generation of reactive oxygen species (ROS) causing cellular toxicity [4,5,6]. Because carbon nanomaterials are often synthesized in the presence of metallic catalysts, heavy metal residues can additionally affect cellular processes as has been shown for carbon nanotubes [7,8].

Studies regarding the toxicology and safety of nanomaterials are often complicated by a lack of comparability partly due to an unnecessary neglect of standardization as well as shortcomings in the physico-chemical characterization of the investigated materials [9]. Additionally, well-established biological testing methods are sometimes compromised by interactions with nanomaterials [10]. This is especially true for more involved test setups based on eukaryotic cells, tissues and animal models where great controversy exists regarding the inherent toxicity of nanomaterials. Hence, investigating toxicity for bacteria offers a welcome simplification of existing model systems, which allows a better control and more facile investigation of bio–nano interactions. However, simplified bacteria systems are not always proper indicators of toxicity in more complex systems [11]. Monitoring bacteria viability is of special importance for assessing the environmental impact of carbon nanomaterials [12]. In their review paper, Mauter and Elimelech discuss the implications of several environmental applications for carbon nanomaterials [13]. In the same vein, carbon nanomaterials can be potentially utilized for disinfection and microbial control [14], for the development of novel antibacterial materials like antibacterial graphene-based paper [15], antibacterial fabrics [16,17], antibacterial wound healing materials [18] or other multifunctional materials [19].

## 2. Inherent Antibacterial Properties of Carbon Nanomaterials

**Figure 1:**
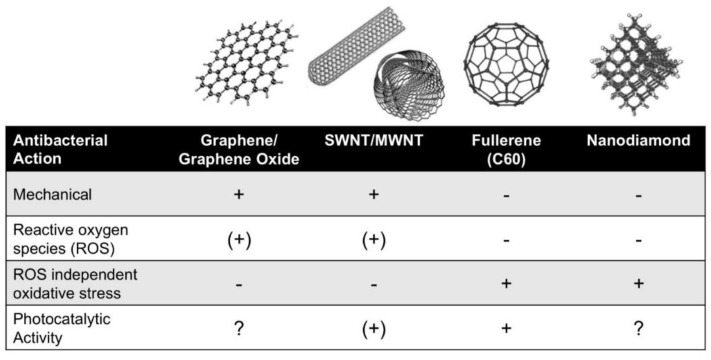
The different allotropes of nanocarbon and their antibacterial action (adapted from [13], with permission from the American Chemical Society 2008).

A quick literature survey reveals that virtually all carbon nanomaterials are able to show antibacterial properties (Figure 1). However, as will be detailed below, the antibacterial activity of CNMs is strongly dependent on surface chemistry, which determines critical factors such as hydrophobicity or oxidation power. This section deals with the inherent antibacterial properties of CNMs. Here, we include properties that originate from standard pretreatment methods, e.g., the use of oxygenating acids that are sometimes used for purification and, at the same time, alter the surface chemistry of the respective materials. Intentional functionalization by more sophisticated methods, which can be used to tailor antibacterial properties of CNMs, will be addressed in the subsequent section. The dispersion state of the colloidal materials is another critical factor as it directly impacts surface contact and bioavailability for bacteria. The dispersion state is also strongly dependent on pretreatment, as well as the presence of additives that might be introduced to stabilize the usually very hydrophobic CNMs in an aqueous dispersion or in biological medium.

For the described studies, bacterial viability was assessed via the expression of stress-related genes [20] or via the minimum inhibitory concentration based on colony forming units [21,22,23,24]. A direct inter-study comparison of the effect of the nanomaterial on bacteria viability is usually not possible, since biological test systems differed significantly, including nanomaterial dosages and exposure times [10].

### 2.1. Graphene

Graphene is a monomolecular layer of carbon that is linked via sp^2^ bonds. Despite its many interesting properties, individual graphene sheets were first prepared as late as 2003 [25], triggering a vibrant and rapidly developing research field. However, dispersing the extremely hydrophobic material proved to be a challenge. In the last years, several dispersing methods for graphene were developed, e.g., the preparation of aqueous dispersions of graphene oxide sheets from graphite using oxidizing acids [26,27]. The resulting graphene oxide nanosheets exhibit lateral dimensions of up to several micrometers while just being one atomic layer thick. The sheets carry a mixture of oxygen containing surface groups, mainly carboxylic acid. Depending on pretreatment and processing route, including the choice of acids and other oxidizing agents, graphene oxide can show diverging toxicological behavior as was shown by Chng and Pumera for adherent lung epithelial cells [28]. Graphene oxide can be converted back to graphene by reducing agents, for example using hydrazine, or even metal-reducing bacteria from the genus *Shewanella* [29]. However, unless dispersion conditions are not carefully controlled, the chemically converted graphene (also called reduced graphene oxide) is usually no longer stable in aqueous dispersion leading to aggregation and sedimentation [26]. Proper dispersion is of course a critical requirement for systematic bacteria studies.

**Figure 2:**
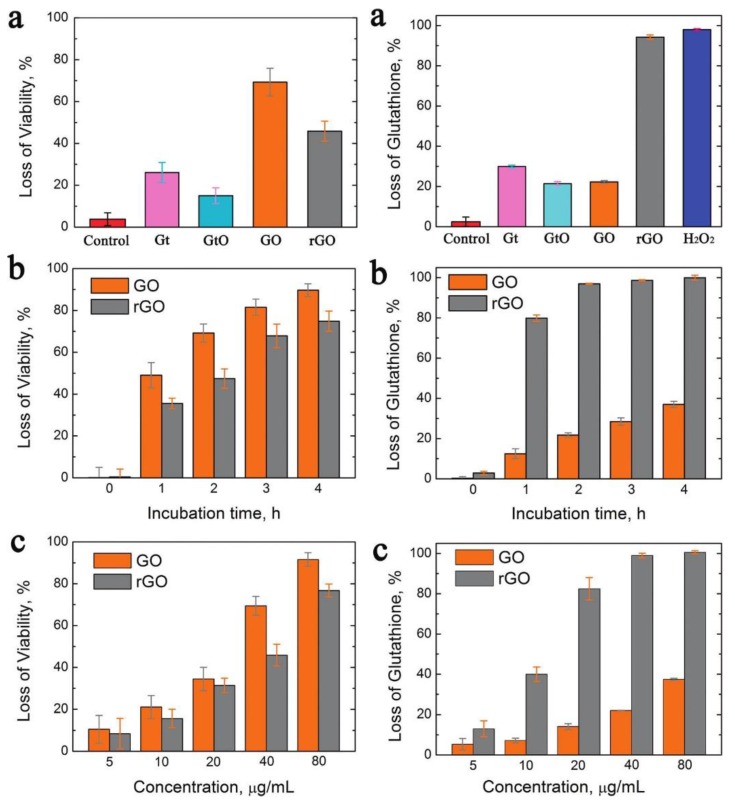
Left: (**a**) Viability of *E. coli* after incubation with Gt (graphite), GtO, GO, and rGO dispersions; (**b**) time-dependent antibacterial activities of GO and rGO with *E. coli*; and (**c**) concentration-dependent antibacterial activities of GO and rGO, *E. coli* was incubated for 2 h. Right: Oxidation of glutathione by graphene-based materials: (**a**) loss of GSH (0.4 mM) after in vitro incubation with Gt, GtO, GO, and rGO dispersions for 2 h; (**b**) time dependent GSH (0.4 mM) oxidation by GO and rGO dispersions (40 μg/mL) after incubation from 0 to 4 h; and (**c**) concentration dependent GSH (0.4 mM) oxidation by GO and rGO after incubation for 2 h. Obtained from [34], with permission from the American Chemical Society 2011.

The antibacterial activity of graphene and graphene oxide is widely documented and has already been utilized to create antibacterial materials for specific applications [15,16,19,30,31,32,33]. Liu et al. compared the antibacterial activity of graphite, graphite oxide, graphene oxide (GO) and reduced graphene oxide (rGO), showing that GO and rGO are both strongly antibacterial (Figure 2) [34]. In these experiments, GO showed stronger antibacterial activity than rGO. Conversely, rGO showed the highest oxidation capacity towards glutathione, which is an indicator for oxidative stress. This discrepancy has been explained by the main antibacterial mechanism of dispersed graphene or graphene oxide which is governed by membrane damage caused by strong dispersion interactions between phospholipids and graphene sheets, which directly relates to the superior dispersion stability of the more hydrophilic GO. In their detailed study, Tu et al., combining both bacteria experiments and simulations, identified two different mechanisms for membrane damage caused by graphene oxide [35]: The first mechanism is based on severe insertion and cutting away of large areas of the cell membrane. The second mechanism is described as a destructive extraction of lipid molecules. Here, phospholipids from the cell membrane spread on partially inserted graphene sheets. An additional mechanical effect was reported by Liu et al., who suggested a size-dependent wrapping of bacteria by graphene oxide sheets that resulted in higher antibacterial activity for larger sheets [36,37]. Using dissipative particle dynamics simulations, Dallavalle and co-authors studied the various potential interactions of graphene with lipid bilayers and emphasized the resulting adverse effects for cell membranes [38].

However, reactive oxygen species were also strongly increased in bacteria affected by graphene and graphene oxide [34,39,40,41]. Increase in ROS was attributed to super-oxide anion-independent oxidative stress on bacterial cells [34]. Oxidative stress is particularly increased when bacterial cells are in contact with conductive reduced graphene oxide and graphite (the latter showing only marginal antibacterial activity) as compared to insulating graphene oxide or graphite oxide. However, since graphene oxide exhibits higher dispersion stability, cell contact is more likely compared to the less stable graphene. Consequently, bacterial death via the membrane damage mechanism, which more strongly depends on well-dispersed nanosheets, dominates the overall antibacterial activity.

The findings for dispersed graphene sheets are confirmed by a study by Akhavan and Ghaderi, who investigated the antibacterial activity of deposited graphene nanowalls, i.e., graphene sheets that were perpendicularly deposited on a substrate [42]. In this respect, Pham et al. found that the antibacterial activity of surface-deposited graphene strongly depends on the density of graphene edges on the surface [43]. In Akhavan’s and Ghaderi’s work, contrary to dispersed graphene nanosheets, rGO shows higher antibacterial activity than GO, since the differences in dispersability are no longer an issue for the surface-deposited nanosheets. Thus, since rGO causes higher oxidative stress than GO, and the mechanical interactions are comparable, rGO shows the stronger effect. The same study demonstrates that gram-positive bacteria are less susceptible to membrane damage than gram-negative bacteria lacking a thick protective peptidoglycan-layer [42].

However, not all studies on interactions of graphene with bacteria show an inhibitory effect. For example, in another study, Akhavan and Ghaderi [44] report that *E. coli* are able to reduce graphene oxide sheets, forming rGO, while significant antibacterial activity occurred only once the graphene sheets have been reduced. In this study, growth of *E. coli* on the GO coated substrate is slightly increased compared to the bare SiO_2_ substrate. This results in a self-limiting growth of bacteria on graphene oxide coated substrates. Note that, in this study, GO was deposited randomly on the substrate without the formation of sharp nanowalls as in the aforementioned study. A similar result is reported by Dellieu et al. who studied CVD grown graphene films on gold and copper substrates. Here, the flatly deposited graphene shows no antibacterial activity of its own and even decreases the antibacterial effect of copper by blocking copper ion flow toward the bacteria [45].

Conversely, Ruiz et al. report that graphene, both in colloidal form and deposited on substrates enhances bacterial growth [46]. In these experiments, precipitation of colloidal GO with bacteria was observed, which seemingly resulted in a growth area for biofilms within the precipitate. However, these findings are merely judged on the basis of measuring the optical density of the dispersion (which should be strongly dependent on the GO concentration [47]) and microscopy. In the context of this study, it should also be noted that testing for bacterial growth inhibition zones on agar plates should only produce negatives (no inhibition) for graphene, since the antibacterial effect is not based on the leaching of soluble species like toxic ions.

In accordance with the previously described study by Akhavan and Ghaderi [44], a slight enhancement of bacterial growth on randomly deposited graphene oxide was reported. The enhanced growth of bacteria on rough (but non-perpendicular) GO films is utilized by Wang et al. to enhance the activity of anaerobic ammonium oxidation bacteria [48].

The apparent discrepancy between biocompatibility and differing antibacterial activities has been explained by Hui et al. [47] In their work, the authors showed that the adsorption of proteins and other biomolecules that are commonly found in nutrient broth on basal planes (i.e., a flat surface) of graphene deactivates its antibacterial activity. However, most antibacterial tests are performed in simple saline solutions that do not contain ingredients that are prone to adsorb on graphene. Consequently, results of the antibacterial activity of graphene (and other nanomaterials [49]) have to be interpreted regarding whether nutrient broths or similar media containing biomolecules that are strongly surface active and readily adsorb on (carbon) nanomaterials were used in the biological experiments. Written concurrently to this overview, these findings on the antibacterial activity have also been very recently summarized in several extensive and detailed reviews [50,51,52].

### 2.2. Carbon Nanotubes

**Figure 3:**
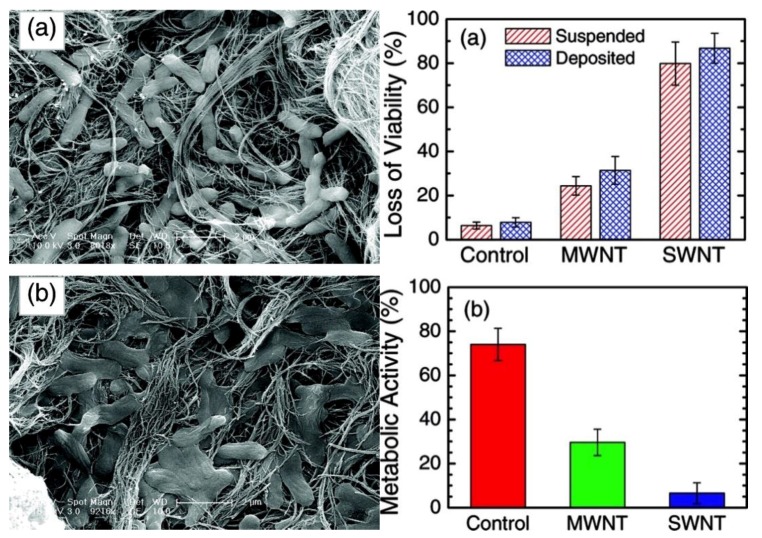
Left: Scanning electron microscopy (SEM) images of *E. coli* cells exposed to CNTs: (**a**) cells incubated with MWNTs for 60 min; and (**b**) cells incubated with SWNTs for 60 min. The bars in both images represent 2 µm. Right: Bacterial cytotoxicity of carbon nanotubes: (**a**) fluorescence-based toxicity assay of SWNTs and MWNTs for suspended- and deposited-type experiments with *E. coli*; and (**b**) metabolic activity test of *E. coli* cells deposited on a CNT-coated filter and a control PVDF membrane. Obtained from [20], with permission from the American Chemical Society 2008.

Carbon nanotubes (CNT) can be described as hollow structures with an extremely high aspect ratio, which are formed by rolled graphene sheets. Depending on the rolling angle, carbon nanotubes can be metallic or semi-conductive. Furthermore, nanotubes are categorized as single-walled nanotubes (SWNTs) and multi-walled nanotubes (MWNTs). The latter consist of several single-walled tubes that are nested inside each other. Carbon nanotubes, especially SWNTs have a significantly higher antibacterial effect than most carbon nanomaterials [12]. The antibacterial activity of purified SWNTs was first demonstrated by Kang et al. [53]. In their detailed follow-up study, Kang et al. show that purified SWNTs and MWNTs seriously impact bacterial membrane integrity upon direct contact. Accordingly, metabolic activity and morphology are compromised as well [20]. They also show that SWNTs exhibit a stronger antibacterial activity than MWNTs, probably caused by their smaller size that facilitates membrane perturbation and provides a larger surface area (Figure 3). Oxidative stress likely plays an additional, albeit minor, role in the antibacterial mechanism [20]. Liu et al. add further detail to the investigation of mechanical effects that govern the antibacterial activity of CNTs [54]. In accordance with the work by Chen et al. [55], they add weight to the hypothesis that SWNT can act as “nano-darts” that pierce bacterial membranes by comparing bacteria with differing membrane robustness and ruling out other mechanisms by performing extensive control experiments. Further studies indicated that MWNTs showed no mutagenic activity in bacteria assays with *E. coli* and *S. typhimurium* [56].

A distinct feature of sp^2^ carbon nanomaterials, including nanotubes, is their special electronic structure causing semi-conductivity, or, in the case of some CNTs, even (pseudo) metallic conductivity. This aspect was investigated by Vecitis et al. [57] who could clearly demonstrate that metallic nanotubes exhibit a much higher antibacterial activity than semi-conducting CNTs. Consequently, electronic effects can also contribute to the antibacterial activity of nanotubes and this might also apply for other carbon nanomaterials. Similar to the effect described in more detail for fullerenes (see below), CNTs can be activated via photosensitization causing the formation of additional ROS [58].

With CNTs, it is especially important to define the dispersion state of the fibrous colloids. Unfunctionalized CNTs are amphiphobic, which means that they are nearly insoluble in most solvents. Accordingly, dispersions of CNTs can show a wide range of aggregation states that define the accessible surface area that might interact with bacteria [59]. Some studies differentiate between CNTs that are deposited on a substrate and dispersed CNTs, showing widely diverging bacteria toxicity (Figure 3) [57], while other studies completely fail to address these critical aspects. Furthermore, toxicological assessments of carbon nanotubes exposures should take into consideration significant presence of catalytically active iron embedded within the nanotubes [60], as well as other byproducts of production or processing. Possible interactions with test systems (e.g., MTT assay) have been reported as well [11]. Finally, CNT dispersions seldom contain unfunctionalized nanotubes. As will be discussed later, surface adsorbed molecules or covalent functional groups significantly alter bacterial reactions.

### 2.3. Fullerenes

Fullerenes are spherical carbon molecules. The most studied fullerene is the Buckminsterfullerene (abbreviated as C_60_), which consists of exactly 60 carbon atoms that are assembled in a pattern resembling a soccer ball. C_60_ is strongly hydrophobic and is only marginally soluble in water. However, C_60_ can be dispersed in water as colloidal aggregates (nano-C_60_ or nC_60_) under various conditions. Consequently, nC_60_ should not be confused with individual C_60_ molecules with diameters of 1 nm. Instead, the colloidal nC_60_ suspension consists of crystalline aggregates with sizes between 25 and 500 nm that are expected to have a different set of properties as compared to bulk C_60_ or individual C_60_ molecules [61]. Although fullerenes are reported as not being very toxic to eukaryotic cells in comparison to nanotubes and other carbon materials [5], they are found to be potent antibacterial agents. Fullerene water suspensions (nC_60_) were tested for antibacterial activity using *B. subtilis* by Lyon et al. [21] This study showed that fractions of nC_60_ containing smaller fullerene aggregates showed greater antibacterial activities. Furthermore, different pretreatment and processing also affects bacteria toxicity [21]. It has been reported that fullerenes adsorb on bacterial membranes [61]. However, in contrast to graphene or nanotubes, fullerenes do not seem to cause alterations to bacterial membranes [21,61,62]. Contrarily to Lyon et al. [63], Fang et al. showed that nC_60_ is able to alter bacterial membrane phase behavior [64]. Although fullerenes can act as an oxidizing agent, Lyon et al. report that the antibacterial activity is not caused by ROS [62]. Instead, fullerenes are able to cause ROS independent oxidative stress, which is the main reason for the antibacterial activity of fullerenes [63]. Accordingly, the antibacterial action of fullerenes is most likely caused by unspecific reactions with membrane proteins and other vital molecules [62]. Note that these effects only emerge in saline media or other minimal buffer systems. If more complex nutrition media are used, the antibacterial activity of nC_60_ disappears, owing to inactivating reactions of media biomolecules with the fullerene surface and enhanced aggregation due to higher ionic strength [65]. Discussing the environmental impact of CNT on the basis of bacteria studies, Lyon et al. assert that fullerenes are toxic to bacteria, but it is difficult to find a realistic test system that does not alter dispersion behavior and thus the toxic response [61].

**Figure 4:**
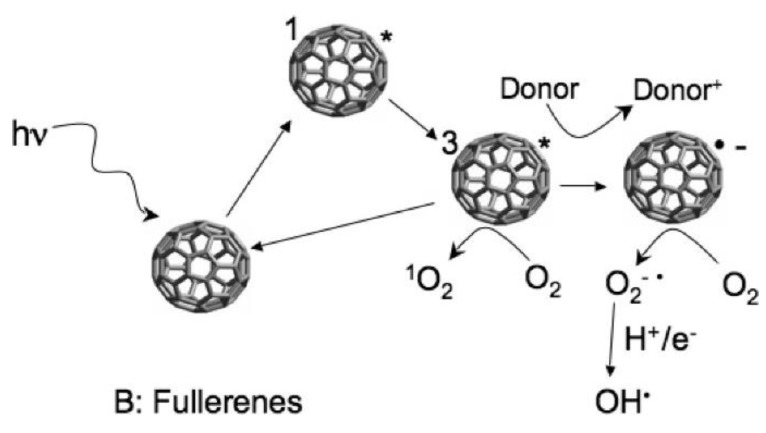
Mechanism of ROS production by fullerenes. Obtained from [69], with permission of the American Chemical Society 2009.

A striking feature of fullerenes is their strong photocatalytic activity, which is caused by their ability to produce oxygen radicals upon UV radiation (Figure 4) [58,66,67]. Photocatalytic activity is well known for semiconducting nanoparticles like TiO_2_. Here, photons with a specific minimum energy (usually within the UV regime) are able to excite electrons into the conducting band creating a conducting electron and an electron hole. The next step after photo-induced charge separation is the transfer of the induced charge to donor or acceptor substrates on the particle surface [68]. In the case of fullerenes, singlet oxygen (^1^O_2_) and superoxide radicals (O_2_^−^) are created at the particle surface (Figure 4) [69]. These oxygen radicals are in turn able to create further ROS and inflict oxidative stress. Brunet et al. compared the photoactivity of fullerenes with that of well-studied TiO_2_ particles [66]. Depending on pre-treatment, not all fullerene water suspensions showed additional bacteria toxicity upon UV radiation, but those that showed this effect did so at much lower particle (fullerene) concentrations than TiO_2_.

### 2.4. Nanodiamond

In contrast to the other carbon nanomaterials, nanodiamond is comprised of sp^3^ hybridized carbon. However, since sp^3^ hybridized carbon would create dangling bonds at the surface, nanodiamond surface chemistry is rich with different oxygen species and residual graphene. The surface chemistry is further enhanced by the faceted nature of the nanocrystals contributing slightly different reactivities at the different crystal planes. The actual composition of the surface chemistry and the amount of defects in the crystal lattice are a result of the synthesis route for nanodiamond, which is either based on detonating TNT and hexagon or similar compounds in a pressurized container (bottom-up) or by milling larger diamonds (top-down). Detonation synthesis yields nanodiamonds with a diameter of about 5 nm, while milled diamonds are usually larger. The final surface chemistry is defined during pre-treatment, which is typically based on various washing steps with oxygenating acids that remove residual graphene. Also in contrast to the other discussed carbon nanomaterials, and as a result of the high amount of oxygen species (mainly carboxyl groups) at the surface, nanodiamond forms very stable aqueous dispersions with high zeta-potentials. However, proper protocols for dispersing individual nanodiamonds have only been established around ten years ago, which explains the comparatively sparse publication record on biological interactions with nanodiamond.

The current (early) consensus on the biocompatibility of NDs is that they are nontoxic for eukaryotic cells [70,71,72,73]. NDs are assumed to have the highest biocompatibility in comparison to all other carbon-based nanomaterials including carbon blacks, single- and multi-walled nanotubes, and fullerenes [74]. Studies on the impact of NDs on small organisms or prokaryotes are rare, but results more recently drifted into a slightly adverse direction. Though Mohan et al. reported the nontoxic nature of NDs with no detectable stress for the worm *Caenorhabditis elegans* [75], Lin et al. assumed that NDs might be more toxic for microorganisms than for animal/human cells. This was tentatively confirmed by investigating the toxic properties of 5 nm and 100 nm NDs on the protozoa *Paramecium caudatum* and *Tetrahymena thermophile*. While smaller NDs were more toxic than bigger particles, carboxylated NDs were less toxic than the non-carboxylated NDs. Although the measured toxicity was relatively low, it was found to be significant [21]. A study on embryonic stem cells was the first hint that NDs might be toxic for eukaryotic cells. Here, carboxylated and oxidized detonation diamonds (4–5 nm) purified by acid treatment were found to induce DNA damage [76]. First studies on the impact of NDs on bacteria were microscopic investigations. For these, high ND concentrations were applied leading to the coverage of bacterial cells [77,78]. Shortly afterwards, Beranova et al. demonstrated the inhibiting effects of detonation ND on *E. coli* growth [79]. The same authors reported that detonation NDs are antibacterial, while larger diamond nanoparticles obtained from milling are not [80]. This study lacked an explanation for the antibacterial properties of detonation ND, but it was suggested that untreated NDs are more effective in killing bacteria than the oxidized form. In our own study [81], which was published around the same time as Beranova’s paper, we found that the antibacterial activity of ND strongly depends on its surface chemistry. While fully carboxylated NDs do not show any antibacterial activity, NDs that are merely partially oxidized show very high antibacterial activity that is similar in potency to silver nanoparticles. Most likely, carboxyl anhydride groups and other reactive oxygen groups on the ND surface are the cause for the strong effect. These findings are somewhat complicated by the fact that the surface chemistry of nanodiamonds is not very well controlled by manufacturers [82], which might explain the differing findings regarding the biocompatibility of ND. Additionally, and analogous to the other described CNMs, we found that the antibacterial effect of ND disappears after contact with biomolecules from nutrition media, most likely due to unspecific reactions with media biomolecules [81,83].

Since nanodiamonds can be semi-conductive as a result of defects in the crystal lattice, NDs have the potential for photocatalytic activity, as could be demonstrated by Jang et al. [84]. However, at the point of writing, an enhancement of the antibacterial effect of ND that is caused by photocatalytic activity has not yet been reported.

### 2.5. Diamond-Like Carbon, Diamond Thin Films

**Figure 5:**
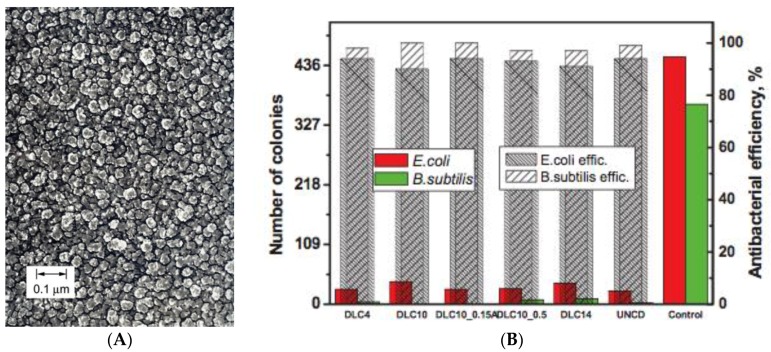
(**A**) DLC film; and (**B**) number of *E. coli* and *B. subtilis* colonies on DLC and UNCD films and the respective antibacterial efficiency. Obtained from [87], with permission of John Wiley & Sons 2013.

Diamond-like carbon (DLC) is a class of carbon coatings. Although they are not colloids per se, the comparison of these coatings with dispersed CNMs is valuable for understanding the antibacterial action of CNMs in general. DLC coatings have attracted great attention from the biomaterials community because they impart biocompatibility, chemical inertness, a low friction coefficient, high hardness, wear and corrosion resistance to medical device surfaces [85,86]. DLC consists of amorphous carbon and/or carbon crystallites and possesses a disordered structure with a mixture of sp^2^ and sp^3^ hybridized carbon [87] (Figure 5a). Depending on the sp^2^/sp^3^ ratio, the properties of DLC can vary significantly. Ultrananocrystalline diamond (UNCD) is very similar to DLC, but has a higher sp^3^ content and distinct nanocrystalline domains.

Both ultrananocrystalline diamond films and DLC exhibit strong antibacterial properties [85,87,88,89] (Figure 5b). Nanocrystalline diamond surfaces with larger crystallite sizes than DLC or UNCD also show bactericidal and anti-adhesive properties [90,91,92], but bacteria toxicity disappears if the crystallites are in the micrometer range [92]. Jelinek et al. compare UNCD and DLC films with varying sp^3^ content showing that all those materials exhibit high antibacterial activity (Figure 4) [87]. Since DLC films are extremely smooth, interfacial roughness or sharp features (as in graphene films) are not important parameters for the antibacterial properties [88]. Instead, the strong hydrophobicity of the coatings might lead to alterations of the cell membrane, resulting in bacterial death [87]. Hydrophobicity and reactivity of the chemically stable coatings also depend on the hydrogen content, with increased hydrogen content resulting in lower antibacterial activity [93]. Various other studies have investigated the interactions of nanocrystalline diamond surfaces with eukaryotic cells and biological molecules [86,91,94,95]. Here, hydrophilic, oxygen-terminated surfaces were described to be the anchor point of interaction on the otherwise anti-adhesive surfaces. Furthermore, diamond thin films were shown to exhibit semiconducting properties [96]. The electrically active surface showed the capacity to form chemical bonds with biomolecules from the surrounding media. Therefore, nanocrystalline diamond surfaces might interact with bacterial cell membranes resulting in an effective membrane-distortion mechanism. This could hinder bacterial adhesion and the subsequent bacterial colonization on the surface [92]. It should be noted that none of the cited antibacterial studies for DLC test for ROS or other oxidative stress that might originate from the redox active properties of the sp^2^ portions of DLC and nanocrystalline diamond.

Furthermore, not all studies for DLC films clearly differentiate between antibacterial and anti-adhesive properties. In this context, antibacterial would mean inhibition of bacterial growth in the surrounding medium, as is known, for example, for copper or silver surfaces through the leaching of toxic ions. Anti-adhesive simply means that bacteria cannot adhere to the surface and thus are not able to form colonies on the material. This can be easily investigated using different methods: for example, the anti-adhesive properties can be tested using stamps that are equipped with the investigated surface. Here, only adherent bacteria are transferred to a nutrition medium and quantified [92]. Conversely, simple incubation tests that monitor the bacteria concentration in a medium containing the investigated material provide information on the antibacterial properties of a (macroscopic) material [87]. Finally, a coating could be antibacterial only to bacteria that adsorb at the surface without significantly influencing bacteria in the surrounding medium, thus featuring a bactericidal surface. This is usually tested by SEM of bacteria on the substrate surface or by applying a small droplet of bacteria medium onto the tested surface followed by incubation over a certain period of time. Afterwards, surviving bacteria are quantified [89]. Differentiating between these different test setups is critical for comparability and general validity of antibacterial tests with macroscopic surfaces, equally important as considering dispersion states in colloidal dispersions of carbon nanomaterials.

As in the case for nanodiamond, it has been shown that DLC can be photocatalytically active [97]. Again, at the point of writing, an enhancement of the antibacterial effect of DLC caused by photocatalytic activity has not yet been reported.

## 3. Functionalization of Carbon Nanomaterials for Tailoring Antibacterial Properties

The body of literature dealing with functionalization, enhancement and tailoring of carbon nanomaterials for different applications is overwhelming. However, since the mechanisms of the antibacterial activity of CNMs are still a point of debate, much fewer publications exist that deal with CNMs that are tailored for enhanced antibacterial properties. The following is a selection of engineered CNM systems with improved antibacterial activity. Next to direct chemical functionalization, CNMs have mostly been enhanced by combining them with other materials, for example in the form of nanocomposites, by loading them with functional biomolecules or by doping diamond.

### 3.1. Graphene

Most publications dealing with antibacterial materials based on graphene describe nanocomposites of graphene with antibacterial nanoparticles or molecules. This work is governed by the principle that the antibacterial activity of the single component is lower than that of the nanocomposites. For example, countless papers describe the formation of antibacterial composites of graphene and silver nanoparticles, improving the antibacterial activity of silver [98,99,100,101,102,103,104,105]. Hybrids of TiO_2_ and graphene have been produced in order to enhance the photocatalytic activity of TiO_2_, which in turn increases the antibacterial activity. Here, graphene serves as electron acceptor, while TiO_2_ is the photocatalytically active component [106]. Work on metal oxide/graphene composites has been summarized in a comprehensive review by Upadhyay et al. [107]. The composite approach has also been demonstrated with polymers. For example, Santos et al. combine graphene with poly(*N*-vinylcarbazole), again resulting in a higher bacteria toxicity than that of the individual components [31]. Furthermore, the biocompatibility of this composite was shown to be higher than that of pure graphene. A similar synergistic increase in antibacterial activity could be demonstrated by loading graphene with antibiotic molecules. For example, Want et al. describe a drug delivery system of graphene and the antibiotic oxide-benzylpenicillin [108], while Abdelhamid et al. describe a similar system for gramicidin [109] and Pandey et al. for gentamicin [110]. While chemical functionalization of graphene is widely investigated, so far, to our knowledge, it has not been applied to enhance antibacterial applications of graphene, although it could be shown, as we already discussed, that different oxidation states significantly alter biological interactions of graphene [28,44].

### 3.2. Carbon Nanotubes

Analogous to graphene, carbon nanotubes have been combined with antibacterial nanoparticles in order to enhance bacteria toxicity of the bare nanoparticles. This has been, for example, demonstrated by Niu et al. for silver nanoparticles [111] and by Akhaven et al. and Woan et al. for TiO_2_ [112,113]. Likewise, work with polymer composites has been done by Joo et al. and Jung et al. who grafted antibacterial polymer Poly[2-(dimethylamino)ethyl methacrylate] onto MWNTs [114,115].

Arias et al. studied the antibacterial effect of –COOH, –OH and –NH_2_ functionalized SWNTs [116]. They note that –COOH and –OH terminated SWNTs are more toxic than ones functionalized with –NH_2_. However, the authors do not characterize the dispersion state or surface charge of the functionalized SWNTs. Accordingly, it is unclear whether the differences in antibacterial activity are merely due to different aggregation states and thus biological accessibility of the nanotubes. The latter might explain the observation that the antibacterial activity is also influenced by the presence of different buffers that might destabilize the nanotube dispersion [116]. A similar argument is brought forth by Su et al. who study the effect on hydroxyl functionalized SWNTs on *P. denitrificants*. They observe an elevated antibacterial activity of the functionalized SWNTs and attribute this increase largely to the enhanced dispersability of the compound [117].

### 3.3. Fullerenes

A rich body of literature exists regarding the organo-chemical functionalization of fullerenes [118]. These fullerene derivates can be equipped with many different functional groups including pyrrolidinium [119], peptides [120] or simple carboxyl and amine terminations [121]. Next to influencing bacteria toxicity, fullerene derivates are known to alter cytotoxicity [122]. In their overview paper, Bosi et al. discuss the biological interactions of fullerenes and fullerene derivates [123]. For example, a higher antibacterial efficacy was observed for positively charged fullerenes in comparison to similar neutral derivatives that even worked against resistant strains of *Mycobacterium tuberculosis* [124]. Similar fullerene derivates that were based on C_60_-bis(*N*,*N*-dimethylpyrrolidinium iodide) were characterized by Mashino et al. [119,125]. These cationic fullerene derivates were found to be both antibacterial and antiproliferative (for different human cancer cell lines), but the antibacterial activity disappeared when long alkyl chains were grafted onto the compounds.

Tang et al. compare four types of fullerenes with different terminations: C_60_, C_60_-OH, C_60_-COOH, C_60_-NH [121]. They find negligible antibacterial effects for C_60_-OH, C_60_-COOH and curiously also for pure C_60_. This discrepancy to the findings discussed above can again be explained by the different dispersion states. The charged derivates will dissolve in water like regular molecules, based on their charged nature, while C_60_ forms aggregates in water, thus decreasing the number of available molecules.

On the other hand, it was shown that fullerols, the fully hydroxylated derivatives of fullerene, show antioxidant properties and thus are able to act as a “free radical sponge” [123]. Accordingly, while fullerol might show some antibacterial activity on its own, it might be shielding from the antibacterial effect of ROS generated by a third material.

The photochemical properties of fullerenes can also be tailored by functionalizing fullerenes. Brunet et al. demonstrate that depending on pretreatment, nC_60_ can be more efficient at generating singlet oxygen and superoxide [66]. For example, Lee et al. synthesized four hexakis C_60_ derivates that all show enhanced photochemical properties as compared to fullerol [126].

### 3.4. Nanodiamond

Similar to fullerenes, one of the main features of nanodiamond is the ease of functionalization of its carboxylated surface [127,128,129]. Even before the inherent antibacterial properties of nanodiamond have been reported, nanodiamond has been functionalized for antibacterial efficacy. This has been achieved by loading nanodiamond with lysozyme, which has been linked either non-covalently to the surface or covalently using a benzoquinone linker [130,131,132,133]. The studies by Mogil’Naya et al. [130,131] show that lysozyme retains its antibacterial activity at the nanodiamond surface, but to a slightly lesser extent than that of free lysozyme. A considerable amount of research has been done in the area of functionalizing nanodiamod with various glycans that are able to disrupt biofilm formation. These findings have been summarized in a comprehensive review by Szunerits et al. [134]. In the same vein, Turchniuk et al. modified nanodiamond with menthol. Here, the menthol modified nanodiamonds are non-toxic to the investigated bacteria, but inhibit biofilm formation more efficiently than dissolved menthol [135].

### 3.5. Diamond-Like Carbon, Diamond Thin Films

In contrast to the sp^2^ materials graphene, nanotubes and fullerenes, one of the main features of diamond coatings is their chemical inertness. Accordingly, modifications of DLC and related diamond thin film materials have been approached by processes stemming from the semi-conductor world. These techniques include plasma treatment [89,136], doping [137,138] and etching [139].

The antibacterial activity of DLC can be modified by the addition of dopants. Shao et al. demonstrate that doping of DLC coatings with a few percent of silicon results in a significant increase of bacterial adhesion compared to pure DLC or stainless steel 316L [137]. The authors attribute this increase to the decrease in total interfacial free energy, which is in turn caused by an increase in the electron donor component of the free energy. The same effect can also be found for nitrogen doped DLC [140].

In a more straightforward manner, DLC can be doped with elements that are toxic to bacteria, like Cu or Ag [138]. Leaching of these toxic ions enhances the antibacterial activity of the modified coatings.

Tong et al. developed a conductive, micropatterned UNCD film that is both biocompatible and inhibits bacterial growth by combining several etching and chemical vapor deposition techniques [139]. The conductive portion of the micropatterned film is based on UNCD, which is doped with nitrogen, thereby increasing the conductivity of diamond by five orders of magnitude.

## 4. Summary and Outlook

This overview clearly shows that all types of carbon nanomaterials have the potential to be antibacterial. However, most significantly, depending on pretreatment, not all CNM samples show antibacterial activity.

A common toxic mechanism that is found in all antibacterial CNMs is the afflicting of oxidative stress to bacteria. This is mainly caused by the redox activity of the sp^2^ portions of the respective materials. However, oxidative stress is seldom the sole or dominating cause for CNM bacteria toxicity. Instead, mechanical interactions (graphene, nanotubes) or unspecific reactivity (fullerenes, nanodiamonds) can play important roles in the toxic mechanism. An interesting feature is the photocatalytic activity of many carbon nanomaterials. While this feature can be exploited in other fields like energy generation or catalysis, it is also a cause for antibacterial activity. However, with the exception of fullerenes and, to a lesser degree, nanotubes, utilizing the photocatalytic activity of CNM for antibacterial activity has not been investigated so far.

Next to pretreatment, the dispersion state is the most critical parameter that needs to be controlled in order to objectively judge the antibacterial activity of CNMs and nanomaterials in general. Dispersion state and reactivity of CNMs can be significantly altered in the presence of media molecules. In fact, most antibacterial effects have been measured in simple saline buffers without any media biomolecules. In the presence of biomolecules, the toxic effect is often strongly diminished or completely disappears.

This raises a question about the reported biocompatibility of CNMs. More complex biological test setups with eukaryotic cells involve complex nutrition media that will strongly diminish the adverse effects that were found in simpler bacteria tests. However, it might be possible that the antibacterial mechanisms that occur in the absence of media are also fundamental mechanisms for cell damage in eukaryotes, but they are not regularly detected in usual experiments because of the presence of media. These effects might resurface under different testing conditions or, worse, in long-time exposure. On the other hand, the presence of bodily fluids could largely negate toxic effects. More toxicological studies are needed to investigate these points. These arguments should also be considered when designing applied materials based on the antibacterial effect of CNMs.
